# Smart Drill for a Streamlined Estimation of the Drilling Angle and Channel Length in Orthopedic Surgical Procedures

**DOI:** 10.3390/bioengineering11060630

**Published:** 2024-06-19

**Authors:** Arsen Ivanišević, Zvonimir Boban, Josip Jurić, Katarina Vukojević

**Affiliations:** 1 Department of Surgery, Division of Orthopaedics and Traumatology, University Hospital of Split, 21000 Split, Croatia; arsenivanisevic@hotmail.com; 2 Department of Medical Physics and Biophysics, University of Split School of Medicine, 21000 Split, Croatia; 3 Independent Researcher, 21000 Split, Croatia; josip.juric92@outlook.com; 4 Department of Anatomy, Histology and Embryology, University of Split School of Medicine, 21000 Split, Croatia; katarina.vukojevic@mefst.hr

**Keywords:** smart drill, screw size, drilling angle, channel length, breakthrough detection, sensor, orthopedic surgery

## Abstract

The estimation of distances and angles is a routine part of an orthopedic surgical procedure. However, despite their prevalence, these steps are most often performed manually, heavily relying on the surgeon’s skill and experience. To address these issues, this study presents a sensor-equipped drill system which enables automatic estimation of the drilling angle and channel length. The angular accuracy and precision of the system were tested over a range of inclination angles and proved to be superior to the manual approach, with mean absolute errors ranging from 1.9 to 4.5 degrees for the manual approach, and from 0.6 to 1.3 degrees with the guided approach. When sensors were used for simultaneous estimation of both the inclination and anteversion angles, the obtained mean absolute errors were 0.35 ± 0.25 and 2 ± 1.33 degrees for the inclination and anteversion angles, respectively. Regarding channel length estimation, using measurements obtained with a Vernier caliper as a reference, the mean absolute error was 0.33 mm and the standard deviation of errors was 0.41 mm. The obtained results indicate a high potential of smart drill systems for improvement of accuracy and precision in orthopedic surgical procedures, enabling better patient clinical outcomes.

## 1. Introduction

Estimating distances and angles is a key part of orthopedic surgical practice. However, despite the high prevalence of these steps in orthopedic surgery, they are still mostly performed manually, heavily relying on the surgeon’s skill and experience. This article addresses issues in manual bone drilling regarding the proper determination of channel length and drilling direction.

Screw length determination is currently performed using a manual depth gauge, which typically consists of a calibrated rod hooked on one end that is manually inserted into the drilled hole to measure its depth. The measuring process is susceptible to human error and varies significantly depending on the surgeon’s experience, differences in technique [1,2,3], and differences in the manufacturer of the depth gauge [2,3]. This can prolong the duration of surgery [4] and lead to complications due to choosing a screw which is either too short or too long [3,5,6,7].

An obvious solution to these problems would be to use fully automatic robotic systems. However, such devices are not widely used due to their high initial cost and specific requirements in terms of staff training and maintenance [8]. Consequently, manual drill systems have been upgraded with specific combinations of current, force, acceleration, and displacement sensors to detect the breakthrough of the second cortex and provide information on channel length [1,9,10,11,12,13,14,15,16,17]. These sensors are either part of completely new drill systems [10,12,18,19] or are modularly designed so they can be attached to existing surgical systems [1].

Another key factor in orthopedic procedures is the surgeon’s ability to determine specific angles accurately and consistently. For example, accurate angle estimation would ensure that the screw direction is always perfectly aligned with the drilling direction, preventing possible screw misplacement. Another example are hip arthroplasty procedures, where precise angle estimates are crucial for optimal patient outcomes. Multiple studies have shown that the estimation of angles using just visual estimates is very inaccurate [20,21,22,23,24,25,26]. Nevertheless, reports on the use of digital tools for angle estimation are scarce and mostly focus on estimating angles restricted to a single plane [25,26,27,28].

In this study, both the channel length and angle estimation issues were addressed by presenting a wireless modular sensor-equipped drill system, which allows for precise and fast estimation of drilling angles and channel lengths. This approach can improve accuracy and precision in orthopedic surgical procedures, thus enabling favorable patient clinical outcomes.

## 2. Materials and Methods

### 2.1. Smart Drill Description

**Figure 1:**
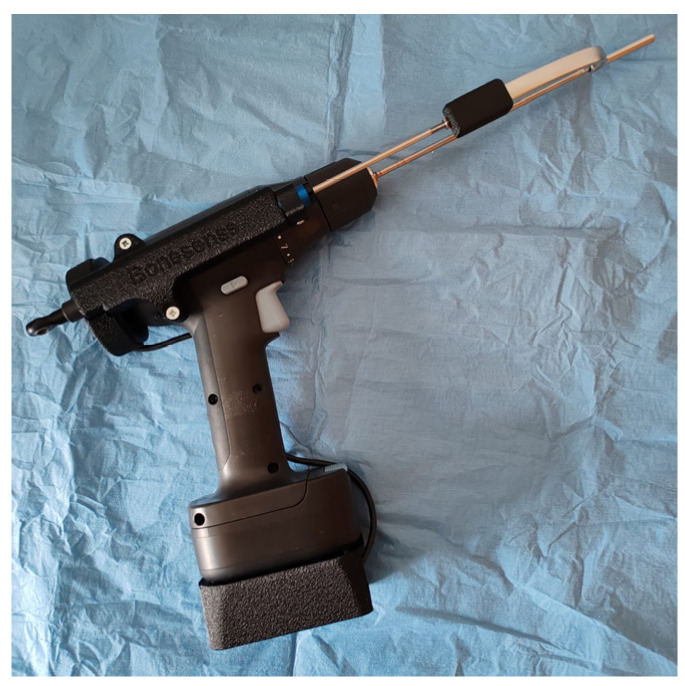
The smart drill system used in the experiments, equipped with an inertial measurement unit and a linear potentiometer.

A cordless power drill (Trixig, Ikea, Sweden) was equipped with an inertial measurement unit (Adafruit BNO085, Ceva, Rockville, MD, USA) and a linear potentiometer (SLS130, Christchurch, Dorset, UK). The sensors were housed in 3D-printed casings, which were then screwed onto the drill (X1-Carbon, Bambu Lab, Shanghai, China) (Figure 1).

### 2.2. Angle Estimation Experimental Setup

**Figure 2:**
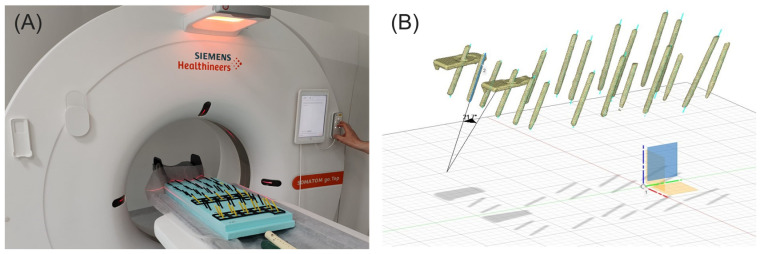
(**A**) CT scanning of the angle estimation experimental setup. (**B**) The obtained 3D model of the rods inserted into the holes to enable angle measurements.

A 3D printer (X1-Carbon, Bambu Lab, China) was used to make the grid for the angle estimation experiments. The grid was attached to a panel made of extruded polystyrene (XPS) and served as a guide to make it easier for the operator to place holes in an organized and tidy manner. The 3D model for angle estimation and comparison to the reference direction was obtained by placing 3D-printed rods into the holes drilled in the XPS and then scanning the surface using computed tomography (CT) (Somatom, Siemens, Munich, Germany) or a 3D scanner (POP 2, Revopoint 3D, Shenzhen, China) (Figure 2A). The obtained model was subsequently imported into a 3D modeling software which was used to calculate the exact angles (Figure 2B).

The number of holes for the angle estimation experiment was estimated based on the study comparing the angle estimation accuracy with and without a digital inclinometer across a variety of angles [25]. With mean and standard values for the inclinometer and manual approach set at 2.5 ± 1 degrees and 8 ± 5 degrees, respectively, we obtained a Cohen’s d of 1.53. Setting the significance level at 0.05 and the desired power at 0.80, we obtained a sample size of 8 per group. Since the cost of adding more samples was low, we conservatively increased this to 10 perforations per group. The sample size calculation was performed using the R programming language [29].

### 2.3. Channel Length Determination Experimental Setup

**Figure 3:**
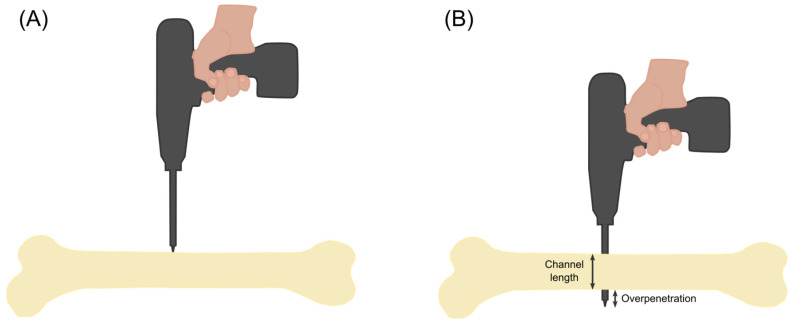
A schematic depiction of the experimental setup for the channel length determination. (**A**) Before the drilling starts. (**B**) After breaking through the second cortex.

An artificial tibia bone (Tibia bone 1149-01, Synbone, Zizers, Switzerland) was used for the channel length determination experiments (Figure 3). To imitate the surrounding tissue, the bone was pressed into a phenol–formaldehyde (PF) foam and kept in place by two clamps on the sides. Ten holes were drilled through the bone and reference values for the channel length were obtained using a Vernier caliper.

**Figure 4:**
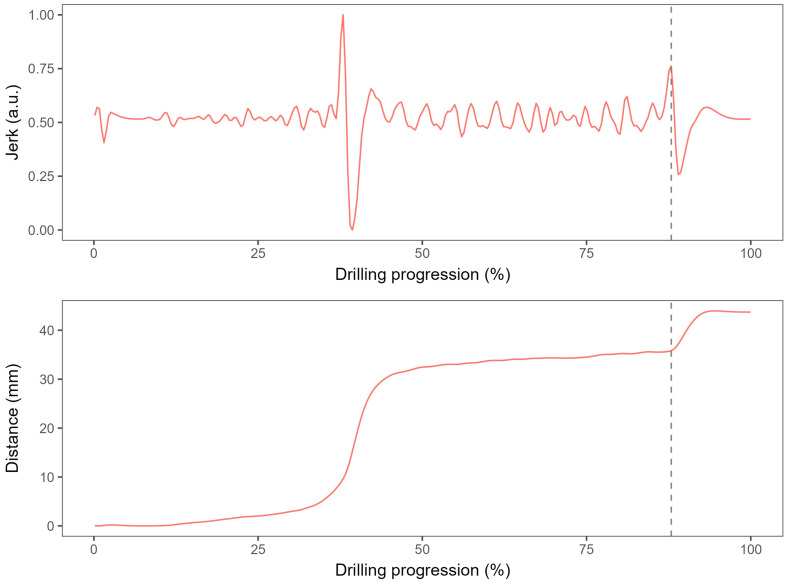
An example of a position signal recorded from the position sensor during drilling and processed in order to obtain the jerk. The vertical dashed line denotes the estimated moment of penetration of the second cortex.

The algorithm for detection of the second cortex position was based on timing the maximum acceleration change (maximum jerk), under the assumption that there will be a change in resistance once the drill bit passes through the second cortex (Figure 4). 

The algorithm can be broken down into four major steps:

1. Load the data from the distance sensor data and apply a low-pass filter to the values to eliminate high-frequency noise;
2. Calculate the acceleration change by numerically differentiating the position data three times using central differences;
3. Filter the data to include only the largest 20% of recorded distances and find the maximum value of the acceleration change in the filtered data;
4. Find the distance at the time of maximum acceleration change to obtain the channel length estimate.

### 2.4. Desktop App for Data Monitoring and Recording

**Figure 5:**
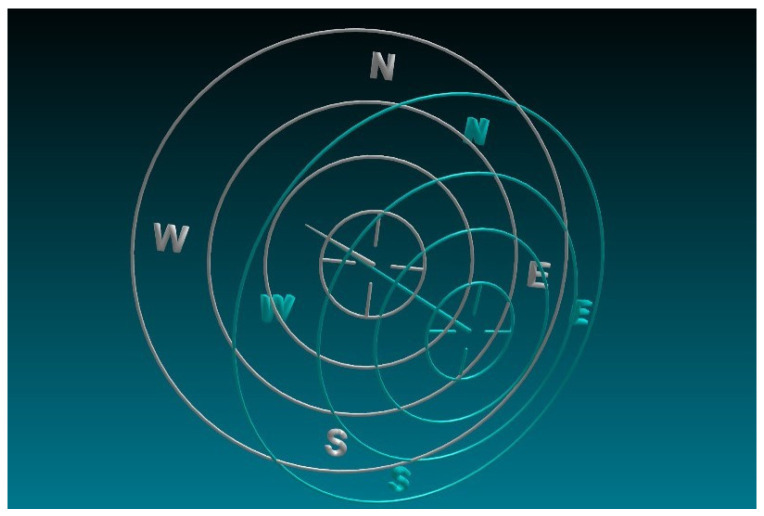
A snapshot of the desktop app for angle navigation and data monitoring. The main panel features two crosshairs, one of which remains stationary and serves as a reference, while the other is moving in response to movement of the drill.

A custom-made app was created in C++ (version 20) to provide visual feedback in angle estimation and store and analyze data. The app displays a crosshair which rotates based on the input from the gyroscopic sensor. When the drill is placed into the housing, the reference direction is remembered by the drill and an additional fixed crosshair is overlayed over the active one to facilitate angle estimation when repositioning the drill for each consecutive hole (Figure 5).

During drilling, the app collects information from the equipped sensors over a wireless network, and then processes it afterward, providing instant feedback regarding the channel length and overpenetration.

### 2.5. Data Analysis and Visualization

Data analysis and visualization were performed using the R programming language [29]. Distribution normality was estimated using the Shapiro–Wilk test and by visually inspecting the Q-Q plots. The performance in terms of angle estimation was assessed through accuracy as a measure of bias, and precision as a measure of consistency. Accuracy was expressed using the mean absolute error defined as the mean absolute deviation from the reference value, and precision as the standard deviation of errors. Written as equations, this translates to

$$Error=x_{i}-reference\;value,$$

$$Mean\;Absolute\;Error=\frac{1}{n}\sum_{i=1}^{n}\left|error\right|,$$

and $SD=Standard\;Deviation=\sqrt{\frac{1}{n}\sum_{i=1}^{n}{error}^{2}}$.

Depending on the distribution normality, Student’s *t*-test or its nonparametric equivalent, the Wilcoxon test, was used to compare the means between two groups. Bartlett’s test was used to test for differences in standard deviations between two groups. A *p*-value lower than 0.05 was deemed statistically significant.

## 3. Results

In order to estimate the performance of the upgraded drill system, two experiments were organized—one to estimate the improvement in drilling angle accuracy and precision, and another to verify the reliability of the channel length estimates. All experiments were performed by an orthopedic specialist with nine years of experience.

### 3.1. Angle Estimation

The aim of the first experiment was to quantify the accuracy and precision in angle estimation. To establish a baseline for comparison, the experiment was first conducted in a traditional manner in which the surgeon determines the angle based on a visual estimate. The same procedure was then repeated with the assistance of mounted sensors to see how great of an improvement was achieved.

The procedure starts with the surgeon inserting the drill bit into a 3D-printed housing to obtain the reference angle, and then drilling ten holes in an XPS panel. For the other part of the experiment, the surgeon is guided by an app collecting data from the sensors on the drill. The app displays a crosshair which rotates based on the input from the sensor. When the drill is placed into the housing, the reference direction is remembered by the drill and an additional fixed crosshair is overlayed over the active one to facilitate angle estimation when repositioning the drill for each consecutive hole (Figure 5).

#### 3.1.1. Angle Estimation for Different Inclination Angles

**Figure 6:**
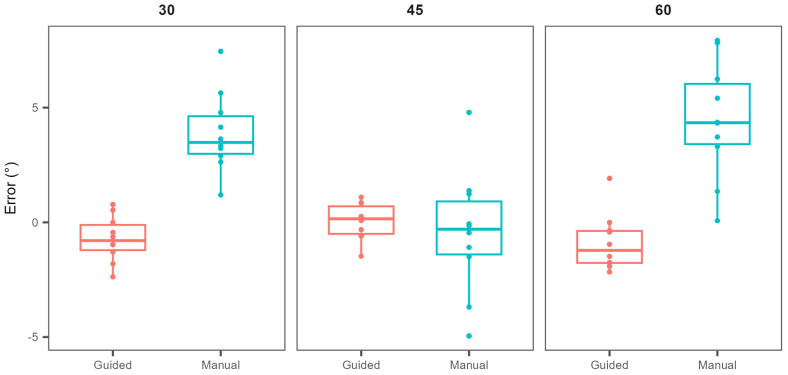
Differences between the measured and reference inclination angles for three reference angle values of 30, 45, and 60 degrees with (Guided) and without (Manual) the assistance of gyroscopic navigation.

The first set of experiments was designed to check how great of an improvement the sensor would introduce when drilling at inclination angles of 30, 45, and 60 degrees. The obtained results indicate an improvement in both accuracy and precision across all three reference angle values (Figure 6 and Table 1).

The mean absolute deviations ranged from 1.9 to 4.5 degrees for the manual approach, and from 0.6 to 1.3 degrees with the guided approach (Table 1). The *p*-values testing differences in mean absolute deviations for the 30-, 45- and 60-degree angles were 4 × 10^−6^, 0.60, and 5 × 10^−5^ (two-tailed two-sample Student’s *t*-test), respectively. To test for differences in precision, standard deviations were also compared, providing *p*-values of 0.11, 0.001, and 0.042 for the angles of 30, 45, and 60 degrees, respectively.

#### 3.1.2. Simultaneous Estimation of the Inclination and Anteversion Angles

Aside from quantifying the capabilities of the system in terms of estimating angles confined to a single plane, the simultaneous estimation of two orthogonal angles was tested as well. The reference direction was determined using an inclination angle of 45 degrees and an anteversion angle of 20 degrees. This angle combination was chosen because of its common use in hip arthroplasty procedures.

Using the manual estimation approach, the average differences with respect to the reference values for the inclination and anteversion angles were 2.4 ± 1.8 degrees and −1.2 ± 2.2 degrees, respectively. With the guided approach, the mean differences were 0.01 ± 0.44 degrees for the inclination and 2 ± 1.4 degrees for the anteversion angle.

**Figure 7:**
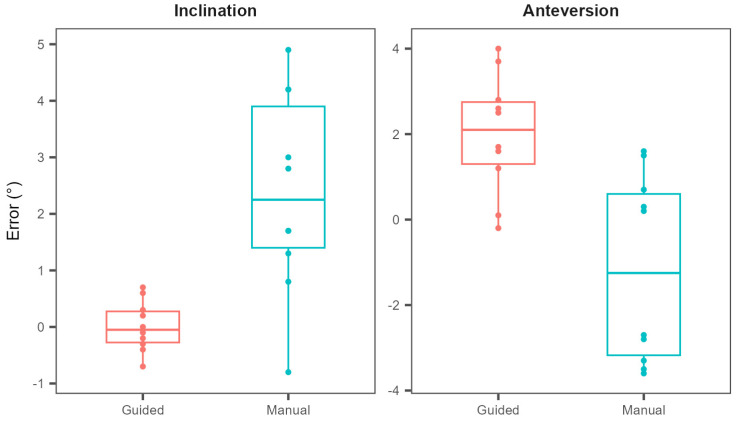
Differences between the measured and reference inclination and anteversion angles with and without the assistance of a motion sensor.

To compare the accuracies between the two approaches, the mean absolute errors between the measured and reference angles were compared (Figure 7). The results for the manual approach were 2.5 ± 1.5 degrees and 2 ± 1.3 degrees for the inclination and anteversion angles, respectively. When the drilling was facilitated by the sensor, the obtained mean absolute errors were 0.35 ± 0.25 degrees for the inclination angle and 2 ± 1.33 degrees for the anteversion angle. Comparing accuracies between the manual and guided approach, a statistically significant difference was obtained for the inclination angle (*p* = 0.0012, two-sample two-tailed Student’s t-test), but not for the anteversion angle (*p* = 0.51, two-sample two-tailed Student’s *t*-test).

Aside from the differences in accuracy of angle estimation, Figure 6 also implies a difference in precision of angle estimation with and without the assistance of a sensor. Testing for differences in standard deviations between the two approaches, statistical significance was again obtained for the inclination angle (*p* < 0.001, Bartlett’s test), but not for the anteversion angle (*p* = 0.99, Bartlett’s test).

### 3.2. Channel Length Determination

**Figure 8:**
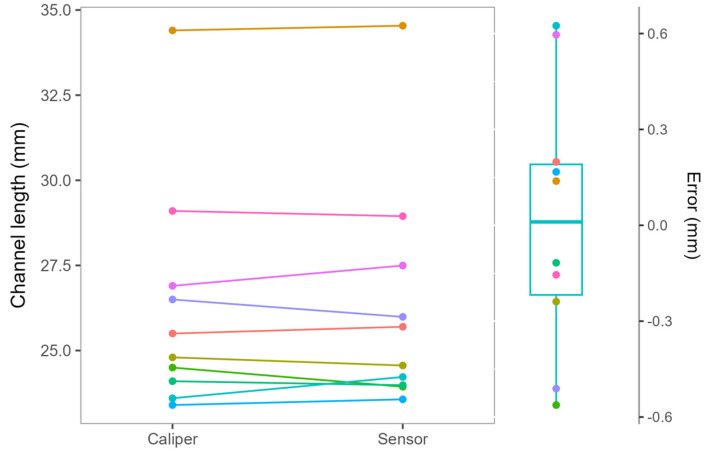
Channel length measurements obtained by analyzing the sensor data (Sensor label) and using a Vernier caliper (Caliper label). The obtained values are presented on the left, with corresponding measurements for each hole connected by a line. A boxplot on the right displays differences between the measurements obtained with the sensor and the caliper. The measurements for each hole were colored the same on both the boxplot and the line chart to facilitate comparison.

To test the quality of channel length measurements using our system, the estimates obtained by analyzing the sensor data were compared with those obtained with a Vernier caliper (Figure 8). The obtained mean difference and standard deviation were 0.01 ± 0.41 mm, and mean absolute error was 0.33 ± 0.21 mm. There was no statistically significant difference between the measurements obtained from the smart drill and the Vernier caliper (*p* = 0.92, paired two-tailed Student’s *t*-test).

Aside from measuring the channel length, the displacement sensor can also provide information on the overpenetration distance due to plunging after breaking the second cortex. The overpenetration value was calculated by subtracting the maximum sensor displacement value from the displacement at the breakthrough detection time. The obtained mean and standard deviation values were 5.5 ± 1.1 mm.

## 4. Discussion

This study presents a wireless modular sensor-equipped drill system, which enables a precise and fast estimation of drilling angle and channel length. The presented results show that the angular accuracy and precision are much better when the surgeon’s estimates are aided by sensors compared to the traditional manual approach. This is in line with previous research on the issue of erroneous angle estimation in orthopedic surgery, reporting angular errors in the range of 5–10 degrees for manual estimation, dropping to approximately 2 degrees with the assistance of tools [20,21,25].

The studies on the use of digital tools for help in angle estimation during surgery are scarce [25,27,28], and even in those studies, estimation is usually provided only for angles confined to a single plane, most often for the inclination angle. This can be due to limitations of the tools, such as in the study by Park et al., where a digital goniometer was used to assess the angle of insertion of Kirschner wires [25], or the nature of the surgical procedure [27].

The angular accuracy and precision of the system were tested over a range of inclination angles and proved to be superior to the manual approach, with mean absolute errors ranging from 1.9 to 4.5 degrees for the manual approach, and from 0.6 to 1.3 degrees with the guided approach. Drilling was also performed in a direction determined by a combination of inclination and anteversion angles, and mean absolute errors of 0.35 ± 0.25 and 2 ± 1.33 degrees were obtained for the inclination and anteversion angles, respectively. The results obtained using a sensor-guided approach were similar or better than those reported in other studies utilizing digital angle measurement tools [20,25,27].

The choice of screw length in orthopedic surgery is most often based on post-drilling channel length measurements obtained using a depth gauge. This process can be very unreliable, with studies reporting average discrepancies between the measured and actual values of approximately 1 to 4 mm depending on the hole geometry and the type of depth gauge used [2]. Another study found that the rate of choosing an overly short screw or a screw which protruded more than 1 mm from the second cortex ranged from approximately 40 to 70% depending on the surgeon’s experience [3]. Aside from enabling a more accurate and precise determination of channel length, using smart drill systems also decreases the duration of surgery by eliminating the use of the depth gauge, with a reported average decrease of 14 s per placed screw [4].

The results presented in this study show an excellent agreement between the measurements obtained by analyzing the sensor data and Vernier caliper measurements. The channel length estimation seems to be both accurate (mean absolute error of 0.33 mm) and precise (error standard deviation of 0.41 mm) enough to be used in a clinical setting. With respect to measurements of overpenetration due to plunging, the obtained mean value was 5.5 ± 1.1 mm. This is in line with results from other studies utilizing freehand drilling, reporting mean values of 6.3 mm [30] and 6.1 mm [4].

Although there are other smart drill systems on the market featuring automatic channel length calculation, they require procurement of a completely new drill system, whereas our system is modular and could be installed on preexisting drill systems. Furthermore, while our proposed system transmits data wirelessly, some of those systems require a wired connection to communicate sensor data to the computer [10,18], which can hinder the surgeon’s motion during surgery.

The limitations of the study are the use of artificial bones instead of human ones, and the estimation of performance by only a single experienced orthopedic surgeon. Using a real human bone should not affect the results much, since synthetic bones are manufactured to simulate the properties of real ones as closely as possible.

With respect to the number of users, we believe that averaging the measurements of the angular accuracy of more subjects would make the differences between the manual and guided system even more pronounced. This is because we do not expect the accuracy to drop when using the guided system, but assume that the accuracy of the manual approach would increase in the general surgeon population. The assumption is based on studies assessing the angular accuracy of surgeons with average results in the range of 5–10 degrees [20,21,25], which is much larger than the average accuracy of approximately 2 degrees obtained by the tested surgeon.

To sum up, studies involving the use of handheld smart drills are very scarce, and address solely either the channel length estimation issue or the problem of inaccurate angle estimation. To our knowledge, this is the first study that combines both features in a single system. The smart drill system described in this study significantly increases the accuracy of angle and channel length estimation compared to the traditional manual approach. Its comparative advantages are its modular design and wireless communication with the computer, allowing installation on preowned surgical drills and making the tool easier to handle.

## 5. Conclusions

This study presents a smart drill system which combines accelerometer, magnetometer, gyroscopic, and linear potentiometer sensors to enable automatic estimation of the drilling angle and channel length in orthopedic surgical procedures.

The angular accuracy and precision of the system were tested over a range of inclination angles or using an inclination–anteversion angle combination as a reference. The sensor-guided approach proved to be superior to the manual approach across all tested conditions, with a mean absolute error under 2 degrees.

Regarding channel length estimation, compared to Vernier caliper measurements, the mean absolute error was 0.33 mm, indicating an excellent agreement of the system estimates with the target values.

To our knowledge, this is the first study that combines both the angle and channel length estimation features in a single system. The results concerning the accuracy and precision of those estimates indicate a significant improvement over the traditional manual approach. Compared to existing approaches, the advantages of this system are its modular design and wireless communication with the computer, allowing installation on preowned surgical drills and making the tool easier to handle.

In order to further explore the system capabilities, we plan to conduct additional studies involving more surgeons with varying amounts of experience. We also plan to perform a human cadaver study to test the system in a surrounding more similar to the surgical one.

## Figures and Tables

**Table 1:** Mean error and MAEs with their standard deviations across three inclination angles. SD = standard deviation, MAE = mean absolute error.

	Mean Error ± SD (°)		MAE ± SD (°)	
Reference Angle	Manual	Guided	Manual	Guided
30	3.9 ± 1.7	−0.72 ± 0.99	3.9 ± 1.7	0.98 ± 0.69
45	−0.5 ± 2.7	0.04 ± 0.80	1.9 ± 1.9	0.63 ± 0.44
60	4.5 ± 2.5	−0.9 ± 1.2	4.5 ± 2.5	1.27 ± 0.88

## Data Availability

The data and code used in this study are available upon reasonable request from the corresponding author.
